# *QuickStats*: Rate of Visits* to Office-Based Physicians,^†^ By Patient Age and Sex — National Ambulatory Medical Care Survey, United States, 2015

**DOI:** 10.15585/mmwr.mm6639a6

**Published:** 2017-10-06

**Figure:**
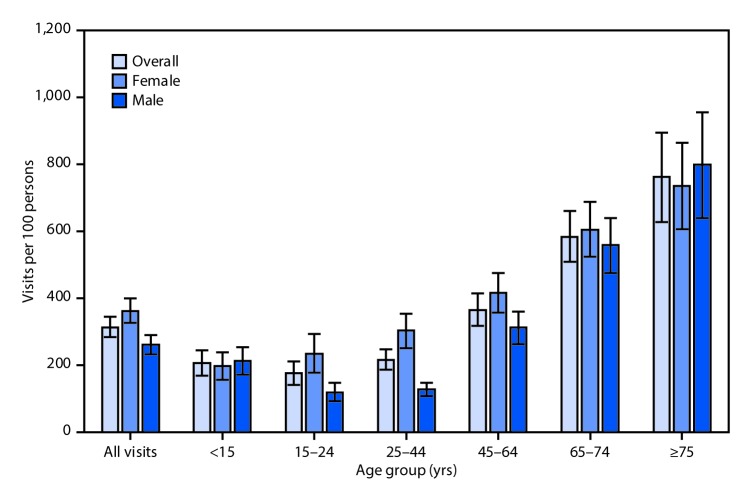
In 2015, the visit rate to office-based physicians was 313 visits per 100 persons. The rate was higher for females (362 per 100) compared with males (262 per 100). For patients in age groups between 15 years and 64 years, the rate for females was higher than the rate for males; for those aged ≥65 years no difference by sex was found. Rates increased with age after the age of 15 years for males and females.

**Source:** National Center for Health Statistics, National Ambulatory Medical Care Survey, 2015. https://www.cdc.gov/nchs/ahcd/ahcd_questionnaires.htm.

